# The Physical Examination Does Matter: A Case of Spontaneous Aortocaval Fistula

**DOI:** 10.7759/cureus.1459

**Published:** 2017-07-11

**Authors:** Rupak Desai, Mikhail Akbashev, Leon Rubinsztain, Andro G Kacharava

**Affiliations:** 1 Research Coordinator, Atlanta Veterans Affairs Medical Center; 2 Department of Medicine, Emory University School of Medicine; 3 Department of Radiology, Atlanta Veterans Affairs Medical Center; 4 Division of Cardiology, Atlanta Veterans Affairs Medical Center

**Keywords:** aortocaval fistula, iliac artery aneurysm, endovascular therapy, physical examination

## Abstract

A spontaneous aortocaval fistula is a rare complication of abdominal aortic aneurysms. In 50 percent of the patients, it presents with the classic signs of a pulsatile abdominal mass, continuous bruit, and low back pain. A high degree of clinical suspicion and a well-performed physical examination are important for its timely diagnosis.

## Introduction

The prevalence of aortocaval fistulas is one percent of all abdominal artery aneurysms [1], but the variety of clinical presentations makes early diagnosis challenging [2-3]. We report a case of a spontaneous iliac artery-to-iliac vein fistula presenting with symptoms suggestive of deep venous thrombosis, recurrent pulmonary embolism, and right heart failure.

## Case presentation

**Figure 1:**
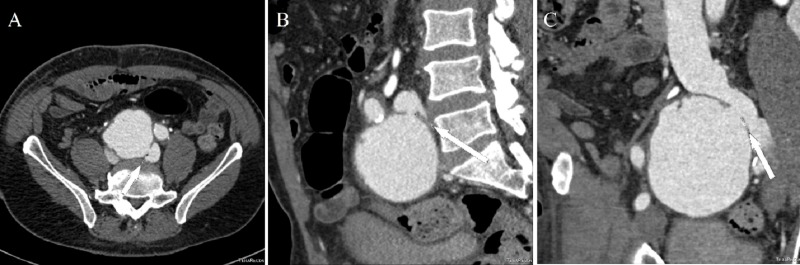
A, B, C Axial, coronal, and sagittal oblique computed tomography angiography (CTA) images through the pelvis demonstrate a fistulous tract (arrows) between an aneurysmal dilatation of the right common iliac artery and the adjacent left common iliac vein with early enhancement of the latter.

A 62-year-old male with hypertension and a smoking history of 50 packs a year was seen in the emergency department, complaining of dyspnea on exertion, fatigue, early satiety, and abdominal discomfort. His blood pressure was 130/70 mm Hg, heart rate was 120/min and regular, lower extremity edema (left more than right) up to his knees was also noted. The cardiovascular exam revealed a 3/6 systolic murmur at the lower-left sternal border, jugular venous distension up to the angle of the mandible, and positive hepatojugular reflux. Cardiomegaly and bilateral pleural effusions were noted in the x-ray. Echocardiography (ECG) showed a normal left ventricle and a dilated, poorly functioning right ventricle. The CT scan ruled out acute pulmonary embolism. The patient had gained 12 lbs since his last hospitalization two months ago when he was diagnosed and treated for a bilateral pulmonary embolism. Since then, he remained on coumadin and his international normalized ratio (INR) was therapeutic. A presumed diagnosis of an acute exacerbation of chronic right ventricular failure was made. Cardiac catheterization revealed nonobstructive coronary artery disease and the left-to-right (L-to-R) shunt Qp/Qs = 3:1, which was initially thought to be at the atrial level. On transesophageal echocardiography, no intracardiac shunt was found. On repeat physical examination, a soft, nontender pulsatile abdominal mass accompanied with systolic bruit was discovered in the right lower quadrant. A computed tomography (CT) angiography revealed a fusiform aneurysm at the level of the distal abdominal aorta and iliac arteries measuring up to 8.0 x 8.5 cm in the greatest transverse dimension complicated by a spontaneous arterio-venous fistula between the right iliac artery and the left iliac vein (Figure 1. A, B, C; white arrows). Exclusion of the right iliac artery aneurysm and fistula closure using the Gore excluder stent (Gore Medical, Arizona, USA) graft were performed. Post-procedure, the patient was discharged in a stable condition with complete resolution of right heart failure symptoms.

## Discussion

The mechanism of the spontaneous formation of an arteriovenous fistula involves the combination of high arterial wall tension and adventitial inflammation, leading to an adhesion and an erosion of the arterial wall into the vein wall [4]. In cases of aortocaval fistulas, the high blood flow and venous hypertension may be responsible for pelvic vein arterialization and engorgement, which can cause asymmetric lower extremity edema [3]. In addition, when complicated by a paradoxical pulmonary embolism, from a mural thrombus [5] originating in an aneurysmal sac, an erroneous diagnosis of venous thromboembolic disease may be made. The paradoxical pulmonary embolism combined with a high pulmonary vascular flow because of the large arterio-venous fistula may lead to the development of pulmonary hypertension complicated by an acute or a chronic right ventricular failure [2].

## Conclusions

A high degree of clinical suspicion and a well-performed physical examination are crucial to the early diagnosis and treatment of aortocaval fistula. The clinical diagnosis can be best supported by a CT angiography, which should be followed by urgent percutaneous or surgical repair.
